# The metabolome of human milk is altered differentially by Holder pasteurization and high hydrostatic pressure processing

**DOI:** 10.3389/fnut.2023.1107054

**Published:** 2023-02-20

**Authors:** Léa Chantal Tran, Lucie Marousez, Marie De Lamballerie, Scott McCulloch, Emmanuel Hermann, Frédéric Gottrand, Delphine Ley, Jean Lesage

**Affiliations:** ^1^Inserm, CHU Lille, U1286 – INFINITE – Institute for Translational Research in Inflammation, University of Lille, Lille, France; ^2^GEPEA, UMR CNRS 6144, ONIRIS, Nantes, France; ^3^Metabolon, Inc., Morrisville, NC, United States; ^4^Division of Gastroenterology Hepatology and Nutrition, Department of Paediatrics, Jeanne de Flandre Children’s Hospital, CHU Lille, Lille, France

**Keywords:** human milk, high hydrostatic pressure, Holder pasteurization, metabolomic, lipids

## Abstract

The milk metabolome is composed of hundreds of molecules that can impact infant development. In preterm infants, sterilized donor milk (DM) is frequently used for their feeding. We aimed to identify differences in the metabolome of DM after two types of milk sterilization: the Holder pasteurization (HoP) and a high hydrostatic pressure (HP) processing. DM samples were sterilized by HoP (62.5°C for 30 min) or processed by HP (350 MPa at 38°C). 595 milk metabolites were analyzed using an untargeted metabolomic analysis. Both treatments differentially altered several classes of compounds. The major changes noted included decreased levels of free fatty acids, phospholipid metabolites, and sphingomyelins. Decreases were more strongly noted in HP samples rather than in HoP ones. Both HoP and HP treatments increased the levels of ceramides and nucleotide compounds. The sterilization of human milk altered its metabolome especially for lipids.

## 1. Introduction

For premature infants, pediatric societies recommend to promote human milk feeding (1–3). However, in some circumstances, mothers of premature infants are not able to provide milk in sufficient quantity due mainly to a foreshortened period of preparatory lactogenesis. When the mother’s own milk is not available, donor milk (DM) is thus the recommended alternative (4). Several hundred human milk banks (HMBs) exist all over the world and provide DM to hospitals (4). In order to ensure the microbial safety of DM, most HMBs currently sterilize human milk using the standard method of Holder pasteurization (HoP) performed by heating milk to 62.5°C for 30 min (4, 5). HoP is widely felt to represent a good compromise between microbiological safety and the nutritional/biological quality of DM, but a growing number of studies show that HoP degrades partly numerous heat-sensitive, bioactive factors such as immunoglobulins, lactoferrin, some vitamins, lysozyme, the bile salt-dependent lipase (BSSL) and some hormones (5–7). Thus, new methods that lead to less deleterious effects on milk compounds are being sought. High hydrostatic pressure (HP) may be an innovative method for the treatment of DM. High pressure is applied in food science, especially to achieve the microbial decontamination of foods while preserving their organoleptic properties for up to 30 years (8). The use of HP to treat DM has emerged recently with data demonstrating that HP maintains numerous bioactive factors such as immunoglobulins, lactoferrin, lysozyme, BSSL enzyme, milk oligosaccharides (HMOs) and several important hormones at levels close to raw milk (7, 9–12). All of these milk factors are well preserved using a HP protocol of four cycles of a moderate pressure (350 MPa) of 5 min each at 38°C previously described by Demazeau et al. (10). However, to date, the effect of HP on other important milk compounds such as metabolites remains unknown.

The human milk metabolome is increasingly studied. It is composed of hundreds of molecules characterized by a low molecular weight of less than 1,500 daltons such as phospholipids, non-esterified fatty acids, amino acids, acylcarnitines and organic acids and intermediates of the tricarboxylic acid cycle, among others (13, 14). Several reports have shown that these metabolites may have a significant impact on infant development (15). As examples, milk biogenic amines were reported to provide protection against infections (16), glutamate has been shown to impact appetite and growth (17), taurine was shown to contribute to neonatal nervous system development (18) as well as creatine (19). Preterm infants are particularly vulnerable due to immaturity of numerous organs. As example, due to immature gut and altered intestinal barrier integrity, these infants are at high risk of developing devastating infections and inflammatory diseases such as necrotizing enterocolitis (NEC) and sepsis (20). It was demonstrated that mother’s milk reduces the incidence of NEC in preterm infants in comparison to the use of milk formula (1–4). Thus, the preservation of all milk compounds including metabolites seems to be crucial for an optimal gut health and development of these infants. In HMBs, due to the recommended sterilization of DM, it can be postulated that slight changes in DM composition after HoP pasteurization may have short-and/or long-term consequences in premature infants. The present study aims at the characterization of the impact of HoP and HP treatment of human milk on its metabolome using an untargeted metabolomic analysis by two separate reverse phase (RP)/ultrahigh performance liquid chromatography-tandem mass spectroscopy (UPLC-MS/MS) methods with positive and negative ion mode electrospray ionization (ESI). Two cohorts of donor milk samples were used in this study. The first one was composed of eight pooled raw milk samples to reduce the variability between samples and, the second one was composed of individual milk samples obtain from 10 donor mothers.

## 2. Materials and methods

### 2.1. Human milk samples

Frozen DM samples from 21 donors were provided by the regional HMB (Lactarium Régional de Lille, CHU Lille). Written informed consent of each donor mothers for the use of their breast milk for research studies was obtain by our HMB and validated by the Lille Hospital [authorization number DOC/LAC/009–2012, Lactarium Régional de Lille, Hôpital Jeanne de Flandre, supervisor: Dr. Véronique Pierrat (MD, PhD)]. To study the effects of HoP and HP treatments, two series of milk samples were used. In the first cohort, we pooled milk samples from 11 women. As previously described (7), after thawing of individual milk samples, 8 different batches of DM were created by mixing various volumes (from 10 to 30 mL) of all DM samples, primarily in order to homogenize DM composition between batches. Then, three aliquots of DM were prepared for each batch: one fraction was stored at −80°C without any other treatment (raw milk); one fraction was subjected to HoP (HoP-DM) according to the standard pasteurization protocol (62.5°C for 30 min) in our HMB and the last fraction was subjected to HP processing as previously described (HP-DM) (7, 10). The set of HP parameters was: 4 cycles of 5 min of a pressure of 350 MPa and at a temperature of 38°C. The second cohort of DM samples used in this study was individual DM samples from 10 donors. Each individual sample was treated similarly by HoP and HP processing. All milk fractions were kept at −80°C until analysis.

### 2.2. Sample preparation

All human milk samples were prepared by Metabolon, Inc. as previously described (13). Briefly, proteins were precipitated with methanol. After removal of organic solvent, sample extracts were stored under nitrogen before analysis. The resulting extract was analyzed on UPLC-MS/MS system using both negative and positive ESI modes.

### 2.3. QC

Several controls were analyzed in concert with the experimental samples: a pooled matrix sample generated by taking a small volume of each experimental sample which served as a technical replicate throughout the data set; extracted water samples served as process blanks; and a cocktail of QC standards were spiked into every analyzed sample, allowed instrument performance monitoring and aided chromatographic alignment.

### 2.4. Ultrahigh performance liquid chromatography-tandem mass spectroscopy (UPLC-MS/MS)

All methods utilized an ACQUITY ultra-performance liquid chromatography (UPLC) (Waters, Milford, MA, United States), a Q Exactive high resolution/accurate mass spectrometer interfaced with a heated electrospray ionization (HESI-II) source (Thermo Scientific, Waltham, MA, United States) and Orbitrap mass analyzer operated at 35,000 mass resolution (21). All samples were treated as previously described for these analyses (13). The MS analysis alternated between MS and data-dependent MSn scans using dynamic exclusion. The scan range covered 70–1,000 m/z.

### 2.5. Data extraction, compound identification and metabolite quantification

All metabolomics analysis from sample treatment, mass spectrometry analysis and data treatment was performed by Metabolon (Morrisville, NC, United States) as previously described (13). Metabolites were identified by automated comparison of the ion features in the experimental samples to the proprietary reference library of chemical standard entries [that included retention time, mass-to-charge ratios (m/z) of the molecule ion, preferred adducts, and in-source fragments as well as associated MS/MS spectra] and curated by visual inspection for quality control using software developed at Metabolon. Identification of known chemical entities is based on comparison to metabolomic library entries of purified standards (>6,000 known compounds). Peaks were quantified using area-under-the-curve. Raw area counts for each metabolite in each sample were normalized to correct for variation resulting from instrument inter-day tuning differences by the median value for each run-day, therefore, setting the medians to 1.0 for each run. This preserved variation between samples but allowed metabolites of widely different raw peak areas to be compared on a similar graphical scale.

### 2.6. Statistical analysis

Statistical analysis was performed by Metabolon as well as using MetaboAnalyst 5.0^1^ for pathway and enrichment analyses. Following log transformation and imputation of missing values, if any, with the minimum observed value for each compound, Welch’s two-sample t-test was used to identify biochemicals that differed significantly between experimental groups, while outliers were identified using the ROUT method. Biochemicals that achieved statistical significance (*p* ≤ 0.05) and demonstrated a low estimate of false discovery rate (*q* > 0.10) were included in this analysis.

## 3. Results

### 3.1. Multivariate analysis of milk metabolic profiles

**Figure 1:**
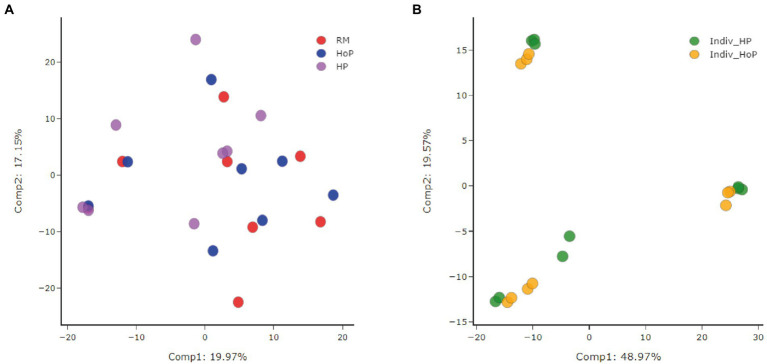
Principal component analysis (PCA) of the two cohorts of DM samples used in this study. The first cohort **(A)** consisted of pooled DM samples (*n* = 8/group) stratified according to treatments [raw milk (RM), HoP-treated milk (HoP) and HP-treated milk (HP)]. No separation of the raw milk, HoP, or HP samples was observed in this first cohort. The second cohort **(B)** consisted of individual DM samples from 10 donors that were treated individually by either method of sterilization (Indiv_HoP and Indiv_HP). A separation between groups was observed for subsets with 3 clusters of samples in this second cohort.

Data were analyzed by Metabolon, Inc. and further analyzed using MetaboAnalyst 5.0 for additional statistical and enrichment analysis. A total of 595 compounds of known identity (named biochemicals) were detected and quantified in milk samples. A Principal Component Analysis (PCA) was performed for the assessment of possible clusters or outliers among the cohorts of DM samples used in this study (Figures 1A,B). The first cohort consisted of pooled DM samples (*n* = 8) stratified according to treatment (raw milk (RM), HoP-treated milk (HoP) and HP-treated milk (HP)). The second cohort consisted of DM samples derived from 10 donors that were split and pasteurized by either method (these samples were named ‘Indiv-HoP’ or ‘Indiv-HP’). By principal component analysis, there was little to no separation of the raw milk, HoP, or HP samples in cohort 1 (Figure 1A). In the second cohort, a separation between groups was observed for subsets with 3 clusters of samples that contained both HoP-and HP-treated samples from the same individuals (Figure 1B). This second cohort confirms that biochemicals detected in DM are slightly modulated between donor mothers.

### 3.2. Modulation of metabolites in cohort 1 of pooled DM samples

**Figure 2:**
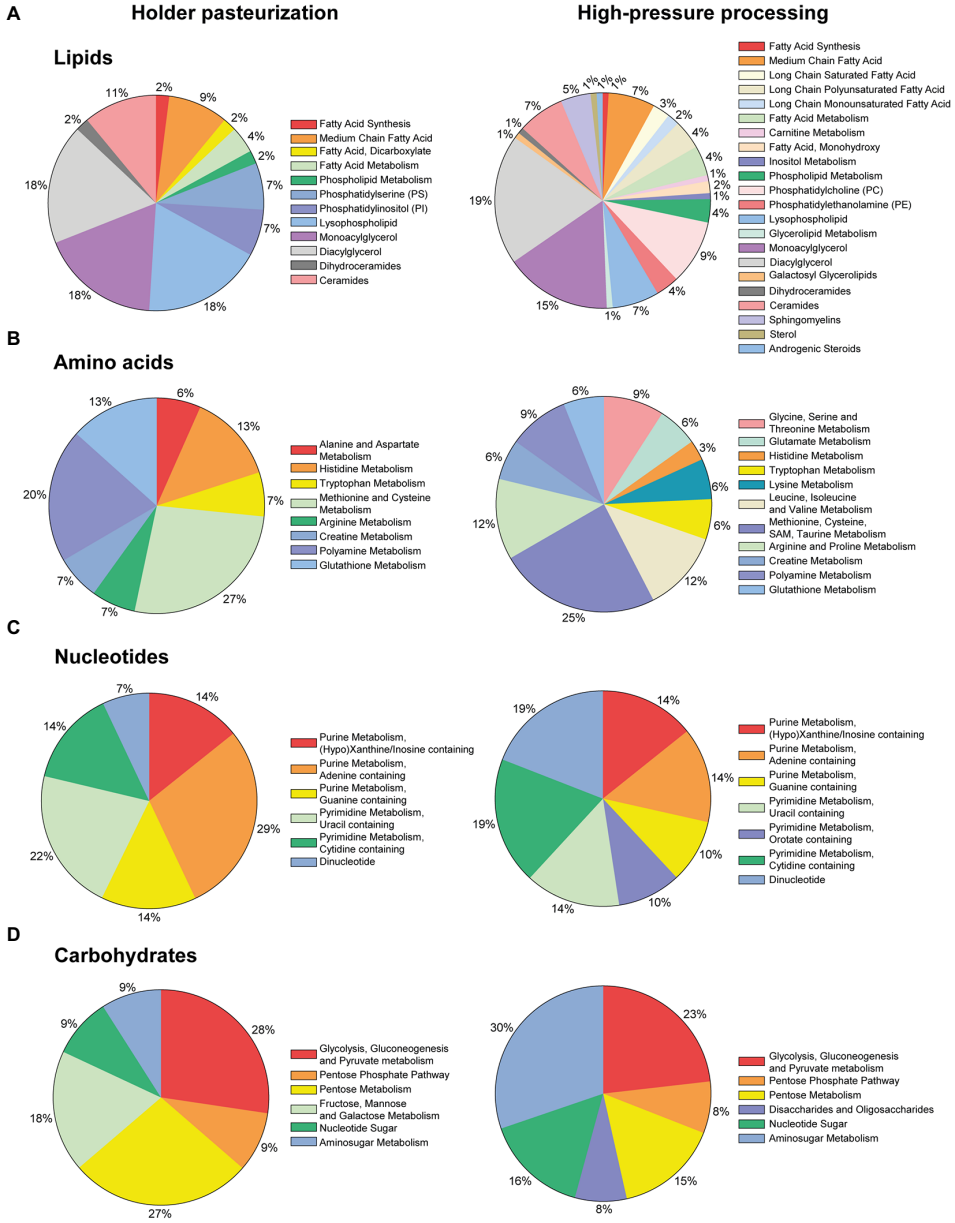
Distribution by sub-classes of the metabolites that were significantly (*p* ≤ 0.05) modulated after Holder pasteurization (HoP) or high-pressure (HP) processing compared to their levels in raw human milk in the first cohort of pooled DM samples (*n* = 8 samples/group).

Statistical analyses to identify overall significant differences (*p* ≤ 0.05) in metabolites between raw-, HoP-and HP-DM samples are shown in Table 1 for the main biochemical classes affected. On the one hand, compared to raw milk, HoP affected significantly 100 milk metabolites. 45% were lipids, 15% were amino acids components, 14% were nucleotides, 11% were carbohydrates. The others remaining metabolites were related to energy (5%), xenobiotics (7%), vitamins and co-factors (3%). On the other hand, HP processing of DM affected a larger number of metabolites (204 compounds). Lipids (55%), amino acids components (16%), and nucleotides (10%) constituted the main affected pathways. Carbohydrates (6%), energy (2%), xenobiotics (7%), vitamins and co-factors (4%) were also slightly affected. Results are summarized in Figure 2 and in Supplementary Tables S1–S5 that described all metabolites that were significantly (*p* ≤ 0.05) modulated after milk sterilization (HoP or HP) in comparison to raw milk (data are expressed as ratio HoP/RM or HP/RM). In this first analysis, four classes of milk biochemicals including lipids, amino acids, nucleotides and carbohydrates constituted the main compounds affected by milk treatment. Interestingly, our data showed that HoP and HP processing differently modulated some of these metabolites.

**Table 1:** Main biochemical class assigned to the identified metabolites that achieved statistical significance (*p* ≤ 0.05) differences between groups in pooled DM samples of cohort 1.

Repeated measures One-Way ANOVA	HoP/RM	HP/RM
Total biochemicals (*p* ≤ 0.05)	100	204
Modulation level (+ /−)	46 / 54	57 / 147
Lipids	17 / 28 (45)	23 / 90 (113)
Amino acids	9 / 6 (15)	7 / 26 (33)
Nucleotides	10 / 4 (14)	15 / 6 (21)
Carbohydrates	5 / 6 (11)	5 / 8 (13)
Energy	3 / 2 (5)	1 / 2 (3)
Xenobiotics	2 / 5 (7)	4 / 11 (15)
Co-factors & vitamins	0 / 3 (3)	2 / 4 (6)

Pooled DM samples were stratified according to treatments [raw milk (RM), HoP-treated milk (HoP) and HP-treated milk (HP)]. The total number of metabolites modulated is indicated as well as the modulation level (in red: increase; in green: decrease). Statistical comparisons were made between HoP and RM groups (HoP/RM ratio) and between HP and RM groups (HP/RM ratio).

### 3.3. Alteration of lipids by HoP and HP processing

After HoP, some lipids (28 compounds) were significantly reduced compared to raw milk, they included 8 monoacylglycerols (MAGs), 7 diacylglycerols (DAGs), 4 medium chain fatty acids (FAs), and 6 phosphatidyl-serine and phosphatidyl-choline compounds (Supplementary Table S1; Figure 2A). One metabolite of FA synthesis (e.g., malonylcarnitine) and two of FA metabolism (palmitoylcarnitine and 3-hydroxybutyrylcarnitine) were also reduced. In an opposite way, eight lysophospholipids, six ceramides and 3 others metabolites (azelate, glycerophosphoserine, linoleoyl-arachidonoyl-glycerol) were found to be increased (Supplementary Table S1).

After HP processing of DM, a higher quantity of lipids (90 compounds) were reduced (Supplementary Table S1; Figure 2A). The main affected metabolites included (but were not limited to) DAGs (21 metabolites), MAGs (18), phosphatidylcholine and phosphatidylethanolamine (15) as well as some medium chain FAs (8), lysophospholipids (5) and sphingomyelins (5). In contrast, 23 metabolites were increased in HP-DM and the most affected class of lipids was ceramides (9 metabolites were increased).

### 3.4. Alteration of amino acid compounds by HoP and HP processing

After milk pasteurization (HoP), six metabolites related to the metabolism of several amino acids (AAs) (aspartate, histidine, methionine, arginine, creatine and glutathione) were reduced (Supplementary Table S2; Figure 2B). In contrast, nine metabolites were increased (histidine, tryptophan and cysteine as well as components of the metabolism of methionine, cysteine, polyamine and glutathione).

After HP processing, twice as metabolites (33 compounds) were modified compared to raw milk (Supplementary Table S2; Figure 2B). They included biochemicals from the metabolism of 11 AAs: glycine, serine, threonine, glutamate, tryptophan, valine, methionine, cysteine, taurine, arginine and proline. Only five AAs metabolites including histidine, lysine, methionine, spermidine and spermine were increased. Isoleucine and arginine levels were also increased.

### 3.5. Alteration of nucleotide compounds by HoP and HP processing

After milk pasteurization (HoP), four metabolites related to the metabolism of purines were decreased (hypoxanthine, adenosine, N1-methyladenosine and N2,N2-dimethylguanosine) whereas 10 compounds from both purines and pyrimidines metabolism pathways were increased (Supplementary Table S3; Figure 2C). They included for purines: allantoin, adenine, N6-methyladenosine and guanosine; and for pyrimidines: uridine 5′-monophosphate, uridine, pseudouridine, cytidine 2′-monophosphate, cytidine and (3′-5′)-guanylylcytidine.

In HP-DM, for these two metabolic pathways, six metabolites were reduced but eight were increased. For purines: AICA ribonucleotide, hypoxanthine and N6-carbamoylthreonyladenosine were decreased whereas inosine, adenosine 5′-monophosphate, adenosine 2′-monophosphate, guanosine, and guanine were increased.

### 3.6. Alteration of carbohydrate compounds and others metabolites by HoP and HP processing

Both HoP and HP processing of DM modulated a small number of carbohydrates metabolites (11 and 13 respectively) in a very similar way (Supplementary Table S4; Figure 2D). These compounds were associated to pyruvate metabolism, pentose metabolism, mannose and galactose metabolisms and nucleotide sugar and amino sugar metabolisms. The oligosaccharide 2′-fucosyllactose (2’FL) was slightly reduced by HP treatment. Finally, a limited number of other metabolites related to the biochemical classes assigned to energy, xenobiotics, co-factors and vitamins were also affected. All of these compounds are reported in the Supplementary Table S5.

### 3.7. Modulation of metabolites in cohort 2 vs. cohort 1

Depending on HMB location, DM is sometimes pooled between different donor mothers before HoP whereas, in others, DM from one donor is not mixed with samples from others donors. To confirm our previous results in cohort 1, we used a second cohort of individual milk samples with a slightly larger number of samples (10 in cohort 2 vs. 8 in cohort 1). Milk metabolites are known to present with inter-individual variations. This inter-individual variation was observed in our principal component analysis (Figure 1B). Then, to conclude on the effects of milk treatments on metabolites and in comparison, to cohort 1, we compared for each sample the metabolites levels after HoP and HP processing (expressed as HP/HoP ratio). This ratio was used to compare the two cohorts together.

The main biochemical classes of metabolites that were modulated in the two cohorts are indicated in Table 2. Comparison between cohorts 1 and 2 confirmed that lipids are drastically affected by both HoP and HP processing (e.g., 127 metabolites from lipids are modulated on a total of 195 affected compounds in cohort 2). In cohort 2, similar changes as those found in cohort 1 were also found for metabolites related to amino acids and carbohydrates. Fewer metabolites from nucleotides were affected in cohort 2 (10 compounds) compared to cohort 1 (21 compounds). Results between the two cohorts are reported in Supplementary Tables S6–S8. In cohort 2, robust evidences showed that numerous classes of lipids were altered by both milk treatments. Globally, numerous free FAs (medium and long chain FAs), DAGs, monoacylglycerols, and structural lipids (phospholipids, lysophospholipids, phosphatidylcholine, and sphingomyelins) were reduced. In contrast, ceramides were increased by milk treatments. Due to the important physiological role of free FAs, phospholipids and ceramides in newborn, we focused on the analysis of these lipid compounds in the sections below.

**Table 2:** Differential effect of HoP and HP processing on the human milk metabolome.

Repeated measures One-Way ANOVA / Matched pairs *t*-test	Cohort 1 (Pool) HP/HoP	Cohort 2 Indiv_HP/Indiv_HoP
Total biochemical (*p* ≤ 0.05)	172	195
Modulation level (+/−)	52 / 120	33 / 162
Lipids	23 / 68 (91)	13 / 114 (127)
Amino acids	5 / 13 (18)	5 / 16 (21)
Nucleotides	13 / 8 (21)	4 / 6 (10)
Carbohydrates	5 /11 (16)	4 /11 (15)
Energy	1 / 3 (4)	1 / 3 (4)
Xenobiotics	2 / 12 (14)	3 / 10 (13)
Co-factors & vitamins	3 / 5 (8)	3 / 2 (5)

Main biochemical class assigned to the identified metabolites that achieved statistical significance (*p* ≤ 0.05) differences between HoP-and HP-treatment in the two cohorts of DM samples are indicated. Cohort 1 was composed of pooled DM samples (*n* = 8/group). The second cohort consisted of individual DM samples from 10 donors that were treated individually by either HoP or HP treatment. Modulations of metabolites by milk treatments was expressed as the ration HP/HoP in both cohorts. The total number of metabolites modulated is indicated as well as the modulation level (in red: increase; in green: decrease).

### 3.8. Focus on free fatty acid metabolites

**Figure 3:**
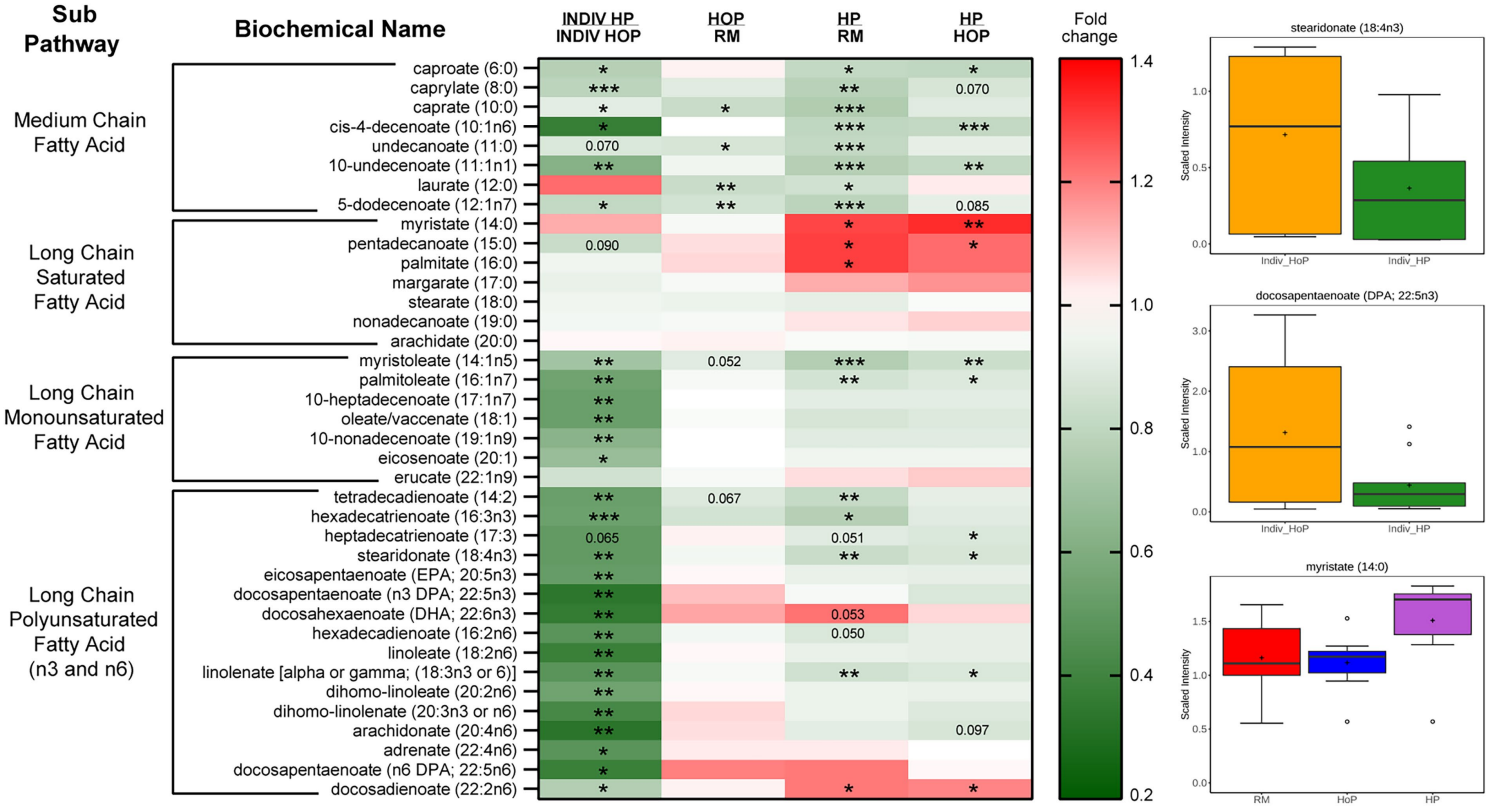
Heatmap (abbreviated) and boxplots of relevant free fatty acids in samples of cohort 1 (pooled RM-, HoP-and HP-samples) and cohort 2 (Indiv_HoP-and Indiv_HP-samples). Metabolites significantly modulated are indicated in colored cells in the table (in red: increase; in green: decrease). Fold-change is indicated in color on the right as well as *p* value in the table (*p ≤ 0.05, **p ≤ 0.01, and ***p ≤ 0.001).

There was a noted decrease in several classes of free FAs (FFAs) in the HP-DM compared the HoP-DM group, particularly significant in cohort 2 (Figure 3). Medium chain, monounsaturated (MUFAs), and polyunsaturated (PUFAs) long chain FFAs showed decreased levels, although interestingly the saturated long chain FFAs did not appear to be affected nearly as much by HoP [stearidonate (18:4n3) and docosapentaenoate (n3 DPA; 22:5n3) are shown]. Within cohort 1, the decreases in fatty acids within the HP-DM samples was evident in both the comparison to raw milk as well as the direct comparison to HoP-DM samples. Interestingly, select saturated long chain FFA [specifically myristate (14:0), pentadecanoate (15:0), and palmitate (16:0)] were found to be increased with the HP treatment in this cohort. Some important FFAs such as DPA, EPA, and DHA, were reduced drastically by HP compared to HoP in cohort 2. Lastly, both MAGs and to a lesser degree DAGs showed a reduced levels in the HP samples in both cohorts demonstrating that HP processing drastically reduced lipids in a deeper way than HoP.

### 3.9. Focus on phospholipid metabolites

**Figure 4:**
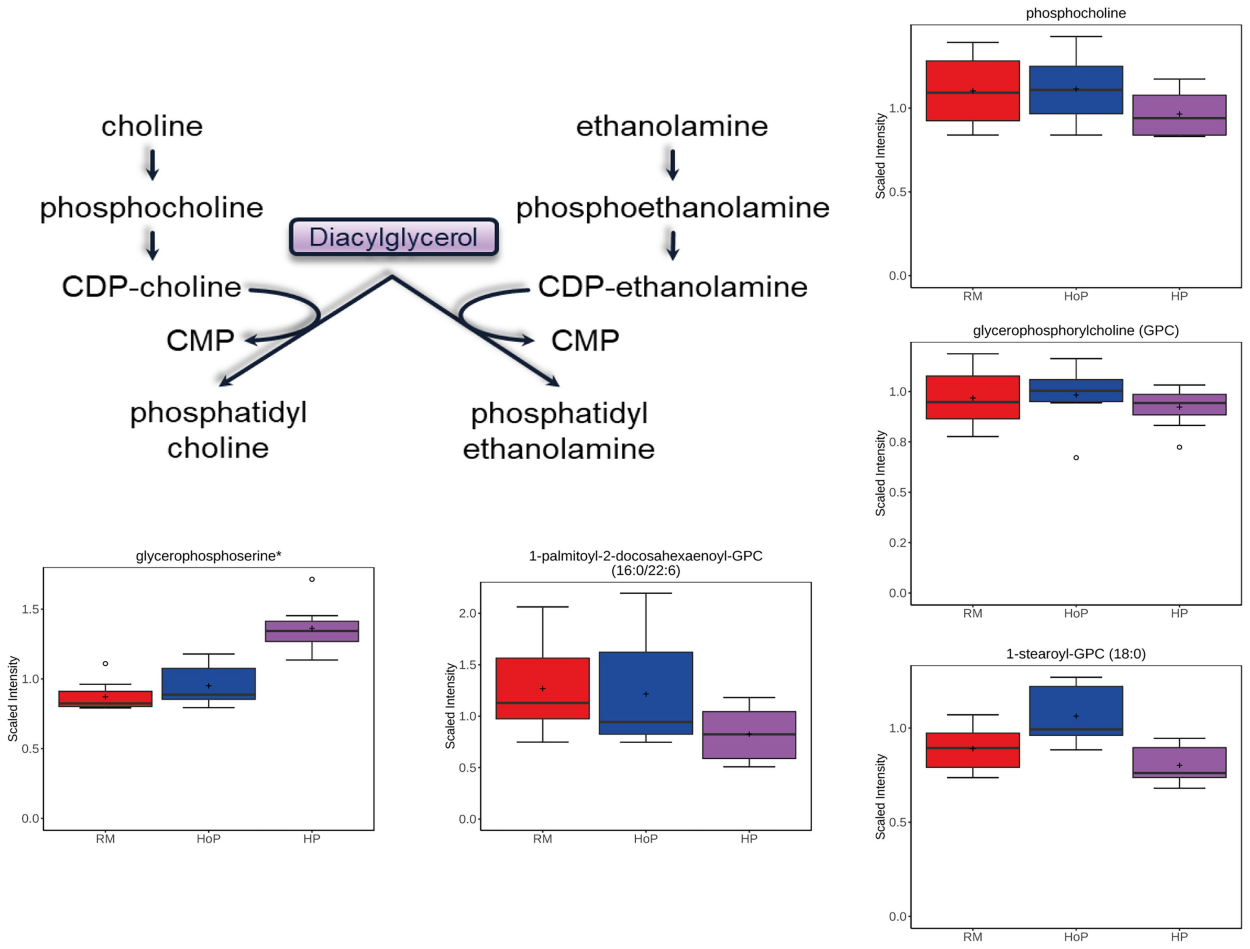
Example of milk metabolites in phospholipid metabolism significantly (*p* ≤ 0.05) affected in cohort 1 by HoP or HP treatment. Phospholipid metabolism schematic diagram is indicated as well as selected boxplots for five metabolites.

Phosphatidylcholine and phosphatidylethanolamine make up the largest proportion of membrane phospholipids. Similar to FFAs, the levels of several phospholipid precursors and the associated lipid chains were decreased after HP processing compared to HoP (Figure 4). This was evident in results of both cohorts of DM samples. These included choline phosphate/phosphocholine, glycerophosphocholine, phosphoethanolamine, glycerophosphoethanolamine, and 1-palmitoyl-2-docosahexaenoyl-GPC (16:0/22:6) (among many other phospholipids). Interestingly, both HP treatment and HoP caused significant increase in glycerophosphoserine, the precursor to the head group of the less prevalent phosphoserine lipids. This could imply that those lipids are more sensitive to both heat and high-pressure treatments. The lysolipids [1-stearoyl-GPC (18:0) is shown in Figure 4], generated from lipase cleavage of phospholipids with the release of a free fatty acid, was also decreased compared to HoP treatment, but compared to RM, HoP caused an increase level suggesting that this cleavage reaction was more sensitive to thermal treatment.

### 3.10. Focus on ceramide metabolites

**Figure 5:**
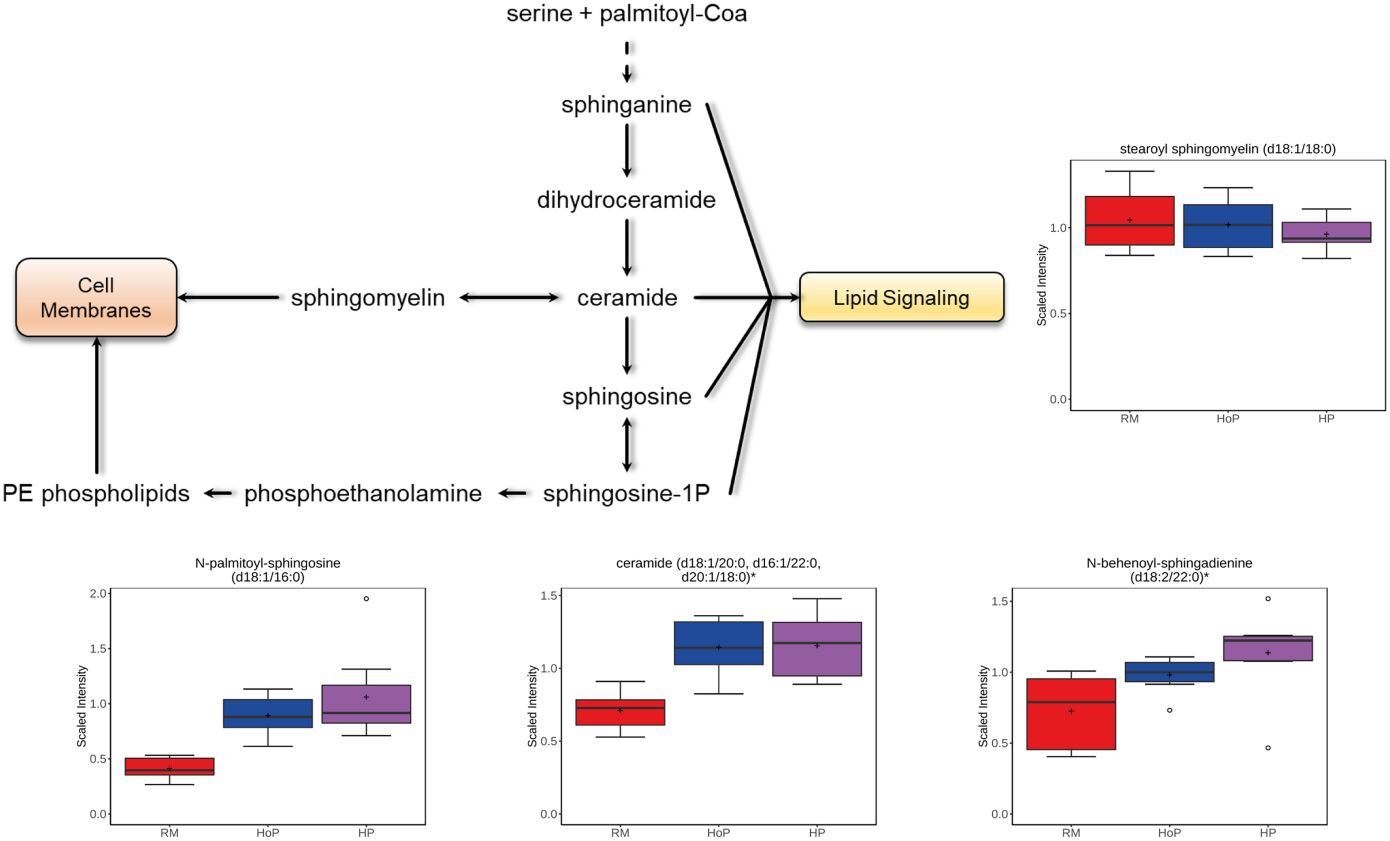
Example of milk metabolites in ceramide metabolism significantly (*p* ≤ 0.05) affected in cohort 1 by HoP or HP treatment. Ceramide metabolism schematic diagram is indicated as well as selected boxplots for four metabolites.

Ceramides are found within cell membrane and act as lipid signaling molecules. In the present study, ceramides, but not hexosylceramides (HCER), lactosylceramides (LCER), or the sphingomyelins, were increased after both HoP and HP processing compared to their levels in raw milk (Figure 5; with ceramides (d18:1/20:0, d16:1/22:0, d20:1/18:0), N-behenoyl-sphingadienine (d18:2/22:0), and N-palmitoyl-sphingosine (d18:1/16:0) which are shown). The majority of sphingomyelins [stearoyl sphingomyelin (d18:1/18:0) is shown] were decreased in HP-DM samples. The conversion of sphingomyelins to ceramide is shown in Figure 5 and may explain the opposite changes of these compounds in treated DM samples.

## 4. Discussion

Human milk is the gold standard for the nutritional support of preterm infants. The alternative for these infants when own mother’s milk is insufficient is to provide sterilized donor milk (DM) instead of infant formula. In this study, we explored the metabolome of DM samples treated by two methods of sterilization, i.e., the Holder pasteurization (HoP) and the use of a high hydrostatic pressure (HP) processing. By an untargeted metabolic analysis, our study demonstrates that treatments of human milk by HoP or HP processing differently altered the composition of milk for several classes of metabolites. Our data clearly demonstrate that lipids are the most affected factors but some changes in metabolites of amino acids metabolism, as well as for nucleotides and carbohydrates ones were also observed.

In human milk, lipids are packaged as milk fat globules, with unique fatty acid (FA) and triacylglycerol (TAG) profiles of a lipid core covered by a membrane mainly composed of polar lipids (22, 23). Phospholipids (PLs) are the main polar lipids in human milk. They include glycerophospholipid (GPL) and sphingolipids. Phosphatidylcholine (PC), phosphatidylethanolamine (PE), phosphatidylinositol (PI), and phosphatidylserine (PS) are the dominant species class of glycerophospholipids, while sphingomyelins (SM) are the dominant species of sphingolipids. Finally, the lysophospholipids (lysophosphatidylcholine (LPC), lysophosphatidylinositol (LPI), phosphatidic acid (PA), and phosphatidylglycerol (PG)) were also identified recently in human milk (24, 25). HoP is used in most HMBs in the world to ensure the microbial safety of DM. However, due to its thermal treatment, an increasing number of studies show that numerous important milk compounds are altered after this pasteurization procedure (5–7). Recently, Ten-Doménech et al. (26) published a very complete study about the effect of HoP on DM lipids by liquid chromatography–mass spectrometry (MS) and gas chromatography-MS analysis. They found that 289 lipids were significantly altered upon HoP pasteurization demonstrating that milk fat globules and their components are very sensitive to thermal treatment. More precisely, these authors found, in accordance with our observations, that multiple lipid classes were reduced by HoP, including DAGs (83% were affected) and TAGs (13% were changed; TAGs are not included in our untargeted dataset), as well as alkenyl-DGs, and linoleic acids and derivatives (26). In comparison, we found that numerous FAs were reduced by HoP including some MAGs, DAGs, medium chain FAs, phosphatidyl-serine and phosphatidyl-choline compounds. In an opposite way, some lysophospholipids and ceramides were found to be increased. As shown in the Figure 5, this latter opposite direction of change may imply the conversion of sphingomyelins to ceramides. Altogether, these data suggest that HoP reduces the nutritional value of DM for lipids. In the literature, conflicting results have been reported (27). However, in a recent study using several hundred DM samples, Piemontese et al. (28) found that HoP reduced macronutrient composition, especially in terms of lipids. This effect was proposed by Vincent et al. (29) to be attributed to the adherence of disrupted milk fat globules to container surfaces and to whether thermal treatment took place in a laboratory environment or in industrial pasteurizers routinely used in HMBs.

High pressure (HP) processing was proposed as an innovative method for the sterilization of DM as it maintains numerous bioactive factors at levels close to raw milk (7, 9–12). However, so far, the effect of HP on the human milk metabolome has never been investigated. In the present study, we used an HP protocol of four cycles of a moderate pressure (350 MPa) of 5 min each at 38°C previously described by Demazeau et al. (10). Using two cohorts of milk samples, our results clearly demonstrate that lipids are the most affected compounds in DM and that HP treatment causes higher changes than HoP for lipids. Indeed, numerous classes of lipids were reduced by HP processing including DAGs, MAGs, phosphatidylcholine, and phosphatidylethanolamine compounds, as well as some medium chain FAs, lysophospholipids, and sphingomyelins. Only a limited number of ceramides were increased in HP-DM. This result suggests that milk fat globules are deeply altered by HP and that in addition several cleavage reactions implicating probably endogenous lipases have altered milk lipids. Zhang et al. (30) have observed an augmented size of milk fat globules between HP-DM and raw DM suggesting an altered stabilization of the milk fat globular membrane after HP processing. The stabilization of fat globules in milk results from steric repulsion by proteins on the surface of milk fat globular membrane, so the disappearance of steric repulsion may lead to the aggregation of milk fat globules and destabilization of the milk colloidal system (31, 32). Several factors could induce this loss of steric repulsion, including a change in fat globule membrane composition, incorporation of milk serum components, enzymatic reactions and changes in the physical environment of the milk fat globule (33). Further studies are thus needed to investigate the precise effect of our HP protocol on milk fat globular membrane composition. Another important effect of HP processing is that this method preserves milk endogenous enzymes activities. Indeed, the moderate pressure used here described by Demazeau et al. (10) was shown by these authors to preserve 95% of the active bile salt-dependent lipase (BSSL) of human milk. Thus, the preservation of probably several active endogenous lipases (from milk serum and from lysed milk cells) by HP processing may explain in part the numerous changes of milk lipids observed in our study after this treatment. Altogether, our data suggest that both milk fat globules as well as lipids are altered by both HoP and HP treatment. In addition to the reduced availability of lipids as general energetic source and precursor material, these changes may also have functional consequences on intestinal development and nervous system maturation in infants. Indeed, both milk lipids and milk globular membrane were shown to be important bioactive factors for intestinal maturation and neurodevelopment (34–37). In addition, as HP processing also reduced several polyunsaturated fatty acids (PUFAs) in our study, their decrease may also alter intestinal epithelial barrier functions as previously demonstrated (36, 37).

Our untargeted metabolomic analysis also showed that others classes of metabolites are slightly affected by milk treatments. They included some amino acids, carbohydrates, and nucleotides, that may have nutritional and functional consequences in infants. As example, we observed augmented nucleotides level in treated samples. We found an increase in the levels of several different nucleotide precursors and other related compounds in both the HoP and HP treated samples in comparison to raw milk. In general, we noted that the HoP treatment causes fewer changes than HP, although it was noted that for some metabolites (5′-CMP, 2′CMP), HoP caused increased levels compared to HP processing. Several of the 5′ and 2′ monophosphate species, in particular, were noted to increase with the treatments, as do some of the free bases (such as guanosine) and dinucleotides [(3′-5′)-adenylyluridine]. It is possible that the elevated heat or pressure of the treatments is causing breakdown of the higher energy di-and tri-phosphate species (i.e., ATP, GTP, etc) due to the labile nature of the phosphodiester bond. Nucleotides account for 2 to 5% of the nonprotein nitrogen fraction of breast milk and provide important cellular, immune, genetic and metabolism functions for the infant (38–40). Thus, the increased levels of these compounds after milk treatment may also have physiological consequences in preterm infants.

## 5. Conclusion

In our study, 595 milk metabolites were analyzed after two procedures of sterilization [Holder pasteurization (HoP) vs. high pressure (HP)]. Both treatments differentially altered several classes of biochemical compounds. The major changes noted included decreased levels of free fatty acids, phospholipid metabolites, and sphingomyelins in the treated samples. In these cases, the decreases were more strongly noted in the HP samples rather than in the HoP group, although differences with raw milk were noted for both treatments. Both HoP and HP treatments caused increased levels of ceramides and some nucleotide associated metabolites. As lipids account for a major source of energy during the fetal/neonatal period and that some lipids also support the development of several organs; further clinical studies are needed to investigate if the altered metabolome of sterilized DM may have nutritional and developmental consequences.

## Data availability statement

The original contributions presented in the study are included in the article/Supplementary material, further inquiries can be directed to the corresponding author. The data presented in the study are deposited in the metabolights repository, accession number MTBLS6980.

## Ethics statement

Written informed consent of each donor mothers for the use of their breast milk for research studies was obtained by our HMB and validated by the Lille Hospital [authorization number DOC/LAC/009-2012, Lactarium Régional de Lille, Hôpital Jeanne de Flandre, supervisor: Véronique Pierrat (MD, PhD)].

## Author contributions

LT, LM, and JL drafted the manuscript. LT, LM, MD, SM, and JL did the experiments and collected the data. MD, SM, EH, FG, and DL reviewed the manuscript. All authors approved the final version before submission.

## Funding

This work belongs to the “HHP-humanmilk” project funded by the French National Research Program AAPG ANR 2018 (number: ANR-18-CE21-0005).

## Conflict of interest

SM was employed by Metabolon (Morrisville, USA).

The remaining authors declare that the research was conducted in the absence of any commercial or financial relationships that could be construed as a potential conflict of interest.

## Publisher’s note

All claims expressed in this article are solely those of the authors and do not necessarily represent those of their affiliated organizations, or those of the publisher, the editors and the reviewers. Any product that may be evaluated in this article, or claim that may be made by its manufacturer, is not guaranteed or endorsed by the publisher.

## Footnotes

^1^https://www.metaboanalyst.ca, accessed date on 6 June 2021
