# Supplementary material for: The metabolome of human milk is altered differentially by Holder pasteurization and high hydrostatic pressure processing
Source: Front Nutr. 2023 Feb 20;10:1107054. doi: 10.3389/fnut.2023.1107054 (PMC9987212; doi:10.3389/fnut.2023.1107054)
Supplement: Supplementary file 1 [file Table_1.docx]

**Table S1.** Milk metabolites in lipids metabolism significantly (p≤0.05) modulated in cohort 1. Eight pooled samples of raw human milk (Raw) and after pasteurization by HoP (HoP) or high hydrostatic pressure (HP) processing were analyzed. Statistical comparisons were made between HoP and RM groups (HoP/RM ratio) and between HP and RM groups (HP/RM ratio). The modulation level is indicated in colored cells (in red: increase; in green: decrease).

| **Sub Pathway** | **Biochemical Name** | **HoP/RM** | **HP/RM** |
| --- | --- | --- | --- |
| **Fatty Acid Synthesis** | malonylcarnitine | **0,68** | **0,79** |
| **Medium Chain Fatty Acid** | caproate (6:0) | **1,02** | **0,81** |
|  | caprylate (8:0) | **0,90** | **0,78** |
|  | caprate (10:0) | **0,83** | **0,75** |
|  | cis-4-decenoate (10:1n6) | **1,00** | **0,80** |
|  | undecanoate (11:0) | **0,87** | **0,81** |
|  | 10-undecenoate (11:1n1) | **0,95** | **0,77** |
|  | laurate (12:0) | **0,83** | **0,85** |
|  | 5-dodecenoate (12:1n7) | **0,86** | **0,79** |
| **Long Chain Saturated Fatty Acid** | myristate (14:0) | **0,97** | **1,29** |
|  | pentadecanoate (15:0) | **1,05** | **1,30** |
|  | palmitate (16:0) | **1,06** | **1,30** |
| **Long Chain Monounsaturated Fatty Acid** | myristoleate (14:1n5) | **0,90** | **0,76** |
|  | palmitoleate (16:1n7) | **0,97** | **0,86** |
| **Long Chain Polyunsaturated Fatty Acid** | tetradecadienoate (14:2) | **0,89** | **0,82** |
| **(n3 and n6)** | hexadecatrienoate (16:3n3) | **0,86** | **0,77** |
|  | stearidonate (18:4n3) | **0,96** | **0,83** |
|  | linolenate [alpha or gamma; (18:3n3 or 6)] | **0,97** | **0,85** |
|  | docosadienoate (22:2n6) | **1,02** | **1,21** |
| **Fatty Acid, Dicarboxylate** | azelate (C9-DC) | **1,78** | **1,14** |
| **Fatty Acid Metabolism** | butyrylcarnitine (C4) | **0,98** | **0,90** |
| **(also BCAA Metabolism)** | propionylcarnitine (C3) | **1,00** | **0,89** |
| **Fatty Acid Metabolism (Acyl Carnitine, SC)** | acetylcarnitine (C2) | **0,96** | **0,86** |
| **Fatty Acid Metabolism (Acyl Carnitine, MC)** | hexanoylcarnitine (C6) | **0,95** | **0,88** |
|  | octanoylcarnitine (C8) | **0,97** | **0,72** |
| **Fatty Acid Metabolism (Acyl Carnitine, LC)** | palmitoylcarnitine (C16) | **0,84** | **1,02** |
| **Fatty Acid Metabolism (Acyl Carnitine, Hydrox)** | (S)-3-hydroxybutyrylcarnitine | **0,90** | **0,97** |
| **Carnitine Metabolism** | carnitine | **0,97** | **0,86** |
| **Fatty Acid, Monohydroxy** | 3-hydroxylaurate | **0,96** | **1,22** |
|  | 3-hydroxymyristate | **1,03** | **1,39** |
| **Inositol Metabolism** | myo-inositol | **0,99** | **0,91** |
| **Phospholipid Metabolism** | choline phosphate | **1,01** | **0,88** |
|  | phosphoethanolamine | **1,02** | **0,91** |
|  | glycerophosphoserine | **1,09** | **1,56** |
|  | glycerophosphoinositol | **1,03** | **1,08** |
| **Phosphatidylcholine (PC)** | 1-palmitoyl-2-palmitoleoyl-GPC (16:0/16:1) | **0,94** | **0,78** |
|  | 1-palmitoyl-2-oleoyl-GPC (16:0/18:1) | **0,97** | **0,86** |
|  | 1-palmitoyl-2-linoleoyl-GPC (16:0/18:2) | **1,00** | **0,85** |
|  | 1-palmitoyl-2-alpha-linolenoyl-GPC (16:0/18:3n3) | **0,82** | **0,63** |
|  | 1-palmitoyl-2-arachidonoyl-GPC (16:0/20:4n6) | **1,04** | **0,69** |
|  | 1-palmitoyl-2-docosahexaenoyl-GPC (16:0/22:6) | **0,93** | **0,66** |
|  | 1-stearoyl-2-linoleoyl-GPC (18:0/18:2) | **0,97** | **0,90** |
|  | 1-stearoyl-2-arachidonoyl-GPC (18:0/20:4) | **0,98** | **0,89** |
|  | 1-oleoyl-2-linoleoyl-GPC (18:1/18:2) | **0,99** | **0,76** |
|  | 1,2-dilinoleoyl-GPC (18:2/18:2) | **0,94** | **0,56** |
|  | 1-linoleoyl-2-arachidonoyl-GPC (18:2/20:4n6) | **0,93** | **0,44** |
| **Phosphatidylethanolamine (PE)** | 1-palmitoyl-2-arachidonoyl-GPE (16:0/20:4) | **0,95** | **0,87** |
|  | 1-palmitoyl-2-docosahexaenoyl-GPE (16:0/22:6) | **0,88** | **0,79** |
|  | 1-oleoyl-2-linoleoyl-GPE (18:1/18:2) | **0,95** | **0,87** |
|  | 1,2-dilinoleoyl-GPE (18:2/18:2) | **0,95** | **0,68** |
| **Phosphatidylserine (PS)** | 1-stearoyl-2-oleoyl-GPS (18:0/18:1) | **0,72** | **1,11** |
|  | 1-stearoyl-2-linoleoyl-GPS (18:0/18:2) | **0,72** | **1,09** |
|  | 1-stearoyl-2-arachidonoyl-GPS (18:0/20:4) | **0,66** | **1,22** |
| **Phosphatidylinositol (PI)** | 1-palmitoyl-2-oleoyl-GPI (16:0/18:1) | **0,81** | **0,95** |
|  | 1-stearoyl-2-oleoyl-GPI (18:0/18:1) | **0,88** | **0,99** |
|  | 1-stearoyl-2-linoleoyl-GPI (18:0/18:2) | **0,89** | **0,99** |
| **Lysophospholipid** | 1-palmitoyl-GPC (16:0) | **2,40** | **1,82** |
|  | 2-palmitoyl-GPC (16:0) | **1,02** | **1,85** |
|  | 1-stearoyl-GPC (18:0) | **1,19** | **0,90** |
|  | 1-oleoyl-GPC (18:1) | **1,81** | **0,69** |
|  | 1-linoleoyl-GPC (18:2) | **1,69** | **0,51** |
|  | 1-palmitoyl-GPE (16:0) | **1,28** | **1,36** |
|  | 1-oleoyl-GPE (18:1) | **1,44** | **0,49** |
|  | 1-linoleoyl-GPE (18:2) | **1,47** | **0,38** |
|  | 1-oleoyl-GPI (18:1) | **1,51** | **0,77** |
| **Glycerolipid Metabolism** | glycerol | **1,08** | **1,23** |
| **Monoacylglycerol** | 1-myristoylglycerol (14:0) | **0,75** | **0,44** |
|  | 1-pentadecanoylglycerol (15:0) | **0,80** | **0,53** |
|  | 1-palmitoylglycerol (16:0) | **0,82** | **0,55** |
|  | 1-palmitoleoylglycerol (16:1) | **0,85** | **0,42** |
|  | 1-margaroylglycerol (17:0) | **0,80** | **0,60** |
|  | 1-oleoylglycerol (18:1) | **0,88** | **0,50** |
|  | 1-linoleoylglycerol (18:2) | **0,93** | **0,46** |
|  | 1-linolenoylglycerol (18:3) | **0,93** | **0,31** |
|  | 2-dihomo-linoleoylglycerol (20:2) | **0,91** | **0,69** |
|  | 1-dihomo-linolenylglycerol (20:3) | **0,97** | **0,45** |
|  | 1-arachidonylglycerol (20:4) | **1,01** | **0,39** |
|  | 1-docosahexaenoylglycerol (22:6) | **1,01** | **0,68** |
|  | 2-myristoylglycerol (14:0) | **0,61** | **0,47** |
|  | 2-palmitoylglycerol (16:0) | **0,61** | **0,59** |
|  | 2-palmitoleoylglycerol (16:1) | **0,64** | **0,21** |
|  | 2-oleoylglycerol (18:1) | **0,78** | **0,41** |
|  | 2-linoleoylglycerol (18:2) | **0,82** | **0,50** |
|  | 1-heptadecenoylglycerol (17:1) | **0,87** | **0,41** |
| **Diacylglycerol** | diacylglycerol (12:0/18:1, 14:0/16:1, 16:0/14:1) [1] | **0,96** | **0,74** |
|  | diacylglycerol (12:0/18:1, 14:0/16:1, 16:0/14:1) [2] | **0,90** | **0,77** |
|  | diacylglycerol (14:0/18:1, 16:0/16:1) [1] | **1,02** | **0,79** |
|  | diacylglycerol (14:0/18:1, 16:0/16:1) [2] | **0,92** | **0,81** |
|  | diacylglycerol (16:1/18:2 [2], 16:0/18:3 [1]) | **0,93** | **0,83** |
|  | myristoyl-linoleoyl-glycerol (14:0/18:2) [1] | **1,00** | **0,87** |
|  | myristoyl-linoleoyl-glycerol (14:0/18:2) [2] | **0,87** | **0,83** |
|  | palmitoyl-palmitoyl-glycerol (16:0/16:0) [2] | **0,73** | **1,72** |
|  | palmitoyl-linoleoyl-glycerol (16:0/18:2) [2] | **0,88** | **0,92** |
|  | palmitoyl-arachidonoyl-glycerol (16:0/20:4) [2] | **0,84** | **0,78** |
|  | palmitoyl-docosahexaenoyl-glycerol (16:0/22:6) [2] | **0,87** | **0,76** |
|  | oleoyl-oleoyl-glycerol (18:1/18:1) [2] | **0,45** | **0,72** |
|  | oleoyl-linoleoyl-glycerol (18:1/18:2) [2] | **0,98** | **0,80** |
|  | linoleoyl-linolenoyl-glycerol (18:2/18:3) [1] | **1,06** | **0,81** |
|  | linolenoyl-linolenoyl-glycerol (18:3/18:3) [1] | **1,01** | **0,68** |
|  | linolenoyl-linolenoyl-glycerol (18:3/18:3) [2] | **1,06** | **0,63** |
|  | stearoyl-arachidonoyl-glycerol (18:0/20:4) [2] | **0,82** | **0,79** |
|  | oleoyl-arachidonoyl-glycerol (18:1/20:4) [1] | **1,02** | **0,77** |
|  | oleoyl-arachidonoyl-glycerol (18:1/20:4) [2] | **0,91** | **0,72** |
|  | linoleoyl-arachidonoyl-glycerol (18:2/20:4) [1] | **1,19** | **0,81** |
|  | linoleoyl-arachidonoyl-glycerol (18:2/20:4) [2] | **1,01** | **0,77** |
|  | stearoyl-docosahexaenoyl-glycerol (18:0/22:6) [2] | **0,87** | **0,80** |
|  | linoleoyl-docosahexaenoyl-glycerol (18:2/22:6) [1] | **1,10** | **0,80** |
|  | linoleoyl-docosahexaenoyl-glycerol (18:2/22:6) [2] | **0,97** | **0,78** |
| **Galactosyl Glycerolipids** | galactosylglycerol | **1,00** | **1,17** |
| **Dihydroceramides** | N-palmitoyl-sphinganine (d18:0/16:0) | **2,01** | **2,45** |
| **Ceramides** | N-palmitoyl-sphingosine (d18:1/16:0) | **2,17** | **2,49** |
|  | N-stearoyl-sphingosine (d18:1/18:0) | **1,62** | **1,63** |
|  | N-palmitoyl-sphingadienine (d18:2/16:0) | **1,06** | **1,96** |
|  | N-behenoyl-sphingadienine (d18:2/22:0) | **1,43** | **1,59** |
|  | ceramide (d18:1/14:0, d16:1/16:0) | **1,83** | **2,56** |
|  | ceramide (d18:1/20:0, d16:1/22:0, d20:1/18:0) | **1,62** | **1,63** |
|  | ceramide (d16:1/24:1, d18:1/22:1) | **1,22** | **1,92** |
|  | ceramide (d18:2/24:1, d18:1/24:2) | **1,07** | **1,60** |
| **Sphingomyelins** | hydroxypalmitoyl sphingomyelin (d18:1/16:0(OH)) | **0,98** | **0,83** |
|  | sphingomyelin (d18:2/16:0, d18:1/16:1) | **1,04** | **0,83** |
|  | sphingomyelin (d18:2/23:0, d18:1/23:1, d17:1/24:1) | **0,97** | **0,87** |
|  | sphingomyelin (d18:1/24:1, d18:2/24:0) | **0,96** | **0,88** |
|  | sphingomyelin (d18:2/24:1, d18:1/24:2) | **0,98** | **0,89** |
| **Sterol** | 4-cholesten-3-one | **0,93** | **0,84** |
| **Androgenic Steroids** | dehydroepiandrosterone sulfate (DHEA-S) | **0,89** | **0,87** |
