# Supplementary material for: The metabolome of human milk is altered differentially by Holder pasteurization and high hydrostatic pressure processing
Source: Front Nutr. 2023 Feb 20;10:1107054. doi: 10.3389/fnut.2023.1107054 (PMC9987212; doi:10.3389/fnut.2023.1107054)
Supplement: Supplementary file 2 [file Table_2.docx]

**Table S2.** Milk metabolites in amino acids metabolism significantly (p≤0.05) modulated in cohort 1. Eight pooled samples of raw human milk (Raw) and after pasteurization by HoP (HoP) or high hydrostatic pressure (HP) processing were analyzed. Statistical comparisons were made between HoP and RM groups (HoP/RM ratio) and between HP and RM groups (HP/RM ratio). The modulation level is indicated in colored cells (in red: increase; in green: decrease).

| **Sub Pathway** | **Biochemical Name** | **HoP/RM** | **HP/RM** |
| --- | --- | --- | --- |
| **Glycine, Serine and Threonine Metabolism** | dimethylglycine | **0,94** | **0,92** |
|  | 2-methylserine | **0,96** | **0,75** |
|  | threonine | **1,01** | **0,92** |
| **Alanine and Aspartate Metabolism** | N-acetylaspartate (NAA) | **0,75** | **0,91** |
| **Glutamate Metabolism** | N-acetylglutamate | **0,93** | **0,86** |
|  | carboxyethyl-GABA | **0,85** | **0,78** |
| **Histidine Metabolism** | histidine | **1,09** | **1,04** |
|  | 1-methyl-4-imidazoleacetate | **0,88** | **0,90** |
|  | 1-methyl-5-imidazoleacetate | **1,13** | **1,27** |
| **Lysine Metabolism** | lysine | **1,04** | **1,65** |
|  | 2-aminoadipate | **0,94** | **0,78** |
| **Tryptophan Metabolism** | tryptophan | **1,10** | **1,08** |
|  | tryptophan betaine | **0,96** | **0,89** |
|  | indoleacetate | **0,78** | **0,69** |
| **Leucine, Isoleucine and Valine Metabolism** | beta-hydroxyisovalerate | **0,90** | **0,84** |
|  | isoleucine | **1,09** | **1,14** |
|  | tiglylcarnitine (C5:1-DC) | **0,82** | **0,77** |
|  | alpha-hydroxyisovalerate | **0,99** | **0,84** |
| **Methionine, Cysteine, SAM** | N-acetylmethionine | **0,90** | **0,85** |
| **and Taurine Metabolism** | N-formylmethionine | **0,91** | **0,85** |
|  | methionine sulfoxide | **1,12** | **1,15** |
|  | S-adenosylmethionine (SAM) | **0,56** | **0,91** |
|  | S-adenosylhomocysteine (SAH) | **0,96** | **0,85** |
|  | cysteine | **1,70** | **0,95** |
|  | S-methylcysteine | **0,99** | **0,91** |
|  | S-methylcysteine sulfoxide | **0,98** | **0,86** |
|  | cysteine s-sulfate | **2,07** | **1,15** |
|  | hypotaurine | **1,06** | **0,89** |
|  | taurine | **0,97** | **0,92** |
| **Urea cycle; Arginine and Proline Metabolism** | arginine | **1,12** | **1,43** |
|  | argininosuccinate | **0,65** | **0,68** |
|  | homoarginine | **0,89** | **0,84** |
|  | trans-4-hydroxyproline | **0,97** | **0,94** |
| **Creatine Metabolism** | creatinine | **0,98** | **0,93** |
|  | creatine phosphate | **0,44** | **0,67** |
| **Polyamine Metabolism** | N-acetylputrescine | **0,94** | **0,86** |
|  | spermidine | **1,29** | **1,36** |
|  | spermine | **1,60** | **1,57** |
|  | 5-methylthioadenosine (MTA) | **2,66** | **1,16** |
| **Glutathione Metabolism** | cysteinylglycine disulfide | **1,24** | **0,76** |
|  | cys-gly, oxidized | **0,58** | **0,27** |
|  | 2-hydroxybutyrate/2-hydroxyisobutyrate | **1,14** | **0,94** |
