# Supplementary material for: The metabolome of human milk is altered differentially by Holder pasteurization and high hydrostatic pressure processing
Source: Front Nutr. 2023 Feb 20;10:1107054. doi: 10.3389/fnut.2023.1107054 (PMC9987212; doi:10.3389/fnut.2023.1107054)
Supplement: Supplementary file 3 [file Table_3.docx]

**Table S3.** Milk metabolites in nucleotides metabolism significantly (p≤0.05) modulated in cohort 1. Eight pooled samples of raw human milk (Raw) and after pasteurization by HoP (HoP) or high hydrostatic pressure (HP) processing were analyzed. Statistical comparisons were made between HoP and RM groups (HoP/RM ratio) and between HP and RM groups (HP/RM ratio). The modulation level is indicated in colored cells (in red: increase; in green: decrease).

| **Sub Pathway** | **Biochemical Name** | **HoP/RM** | **HP/RM** |
| --- | --- | --- | --- |
| **Purine Metabolism,** | AICA ribonucleotide | **1,01** | **0,86** |
| **(Hypo)Xanthine/Inosine containing** | inosine | **1,12** | **1,93** |
|  | hypoxanthine | **0,53** | **0,65** |
|  | allantoin | **1,36** | **1,10** |
| **Purine Metabolism, Adenine containing** | adenosine 5'-monophosphate (AMP) | **0,94** | **1,96** |
|  | adenosine 2'-monophosphate (2'-AMP) | **1,09** | **4,71** |
|  | adenosine | **0,16** | **0,60** |
|  | adenine | **4,27** | **0,96** |
|  | N1-methyladenosine | **0,82** | **0,89** |
|  | N6-methyladenosine | **2,71** | **1,18** |
|  | N6-carbamoylthreonyladenosine | **0,99** | **0,90** |
| **Purine Metabolism, Guanine containing** | guanosine | **2,30** | **6,34** |
|  | guanine | **0,99** | **1,65** |
|  | N2,N2-dimethylguanosine | **0,83** | **1,02** |
| **Pyrimidine Metabolism, Orotate containing** | dihydroorotate | **1,08** | **1,26** |
|  | orotate | **0,97** | **0,92** |
| **Pyrimidine Metabolism, Uracil containing** | uridine 5'-monophosphate (UMP) | **1,43** | **1,66** |
|  | uridine | **1,20** | **1,29** |
|  | pseudouridine | **1,13** | **1,06** |
|  | 3-(3-amino-3-carboxypropyl)uridine | **0,98** | **0,87** |
| **Pyrimidine Metabolism, Cytidine containing** | cytidine 5'-monophosphate (5'-CMP) | **0,90** | **0,54** |
|  | cytidine 2' or 3'-monophosphate (2' or 3'-CMP) | **2,88** | **2,05** |
|  | cytidine | **1,30** | **1,95** |
|  | cytosine | **1,17** | **1,71** |
| **Dinucleotide** | (3'-5')-adenylylcytidine | **1,21** | **2,22** |
|  | (3'-5')-adenylyluridine | **1,36** | **2,52** |
|  | (3'-5')-cytidylyluridine | **1,02** | **1,95** |
|  | (3'-5')-guanylylcytidine | **1,37** | **1,51** |
