# Supplementary material for: The metabolome of human milk is altered differentially by Holder pasteurization and high hydrostatic pressure processing
Source: Front Nutr. 2023 Feb 20;10:1107054. doi: 10.3389/fnut.2023.1107054 (PMC9987212; doi:10.3389/fnut.2023.1107054)
Supplement: Supplementary file 4 [file Table_4.docx]

**Table S4.** Milk metabolites in carbohydrates metabolism significantly (p≤0.05) modulated in cohort 1. Eight pooled samples of raw human milk (Raw) and after pasteurization by HoP (HoP) or high hydrostatic pressure (HP) processing were analyzed. Statistical comparisons were made between HoP and RM groups (HoP/RM ratio) and between HP and RM groups (HP/RM ratio). The modulation level is indicated in colored cells (in red: increase; in green: decrease).

| **Sub Pathway** | **Biochemical Name** | **HoP/RM** | **HP/RM** |
| --- | --- | --- | --- |
| **Glycolysis, Gluconeogenesis,** | 1,5-anhydroglucitol (1,5-AG) | **0,94** | **0,82** |
| **and Pyruvate Metabolism** | dihydroxyacetone phosphate (DHAP) | **0,56** | **0,71** |
|  | pyruvate | **2,96** | **1,19** |
|  | glycerate | **0,46** | **0,97** |
|  | sedoheptulose-7-phosphate | **1,10** | **0,80** |
| **Pentose Phosphate Pathway** | ribonate | **0,28** | **1,03** |
| **Pentose Metabolism** | arabonate/xylonate | **10,31** | **2,33** |
|  | lyxonate | **6,58** | **1,97** |
|  | 2'-fucosyllactose | **0,99** | **0,88** |
| **Disaccharides and Oligosaccharides** | mannose | **0,87** | **0,90** |
| **Fructose, Mannose and Galactose Metabolism** | galactonate | **0,69** | **1,04** |
|  | UDP-N-acetylglucosamine/galactosamine | **0,95** | **0,78** |
| **Nucleotide Sugar** | cytidine 5'-monophospho-N-acetylneuraminic acid | **0,26** | **0,43** |
|  | N-acetylglucosamine 6-phosphate | **0,99** | **0,91** |
| **Aminosugar Metabolism** | N-acetylneuraminate | **2,02** | **1,16** |
|  | erythronate | **0,99** | **0,83** |
|  | N-acetylglucosamine/N-acetylgalactosamine | **1,04** | **1,31** |
|  |  |  |  |
