# Supplementary material for: The metabolome of human milk is altered differentially by Holder pasteurization and high hydrostatic pressure processing
Source: Front Nutr. 2023 Feb 20;10:1107054. doi: 10.3389/fnut.2023.1107054 (PMC9987212; doi:10.3389/fnut.2023.1107054)
Supplement: Supplementary file 5 [file Table_5.docx]

**Table S5.** Milk metabolites in energy, xenobiotics, co-factors and vitamins metabolism significantly (p≤0.05) modulated in cohort 1. Eight pooled samples of raw human milk (Raw) and after pasteurization by HoP (HoP) or high hydrostatic pressure (HP) processing were analyzed. Statistical comparisons were made between HoP and RM groups (HoP/RM ratio) and between HP and RM groups (HP/RM ratio). The modulation level is indicated in colored cells (in red: increase; in green: decrease).

| **Sub Pathway** | **Biochemical Name** | **HoP/RM** | **HP/RM** |
| --- | --- | --- | --- |
| TCA Cycle | citrate | **0,95** | **0,93** |
|  | aconitate [cis or trans] | **2,59** | **1,02** |
|  | alpha-ketoglutarate | **1,26** | **1,11** |
|  | succinate | **0,71** | **1,04** |
|  | fumarate | **1,40** | **1,01** |
|  | malate | **0,90** | **0,88** |
| Nicotinamide Metabolism | quinolinate | **1,12** | **0,54** |
|  | nicotinamide adenine dinucleotide (NAD+) | **0,96** | **0,80** |
| Ascorbate and Aldarate Metabolism | dehydroascorbate | **1,10** | **1,44** |
|  | 2-O-methylascorbic acid | **0,98** | **0,93** |
|  | threonate | **0,48** | **0,54** |
|  | oxalate (ethanedioate) | **0,59** | **1,20** |
| Porphyrin Metabolism | bilirubin (Z,Z) | **0,56** | **0,84** |
|  | bilirubin (E,E) | **0,60** | **0,67** |
| Vitamin A Metabolism | retinol (vitamin A) | **0,97** | **0,87** |
| Benzoate Metabolism | 4-hydroxyhippurate | **0,98** | **0,79** |
|  | benzoate | **1,06** | **1,40** |
|  | catechol sulfate | **0,98** | **0,83** |
|  | guaiacol sulfate | **1,02** | **0,89** |
| Xanthine Metabolism | theobromine | **0,84** | **0,94** |
|  | 3-methylxanthine | **0,97** | **0,87** |
|  | 7-methylxanthine | **0,97** | **0,80** |
| Food Component | 3-hydroxyindolin-2-one | **0,86** | **0,91** |
|  | gluconate | **1,24** | **1,44** |
|  | ergothioneine | **2,74** | **0,99** |
|  | erythritol | **1,00** | **0,87** |
|  | histidine betaine (hercynine) | **1,11** | **2,50** |
|  | tartronate (hydroxymalonate) | **0,82** | **0,56** |
|  | ethyl beta-glucopyranoside | **0,96** | **0,81** |
| Chemical | trizma acetate | **0,94** | **1,08** |
|  | 1,2,3-benzenetriol sulfate (2) | **1,00** | **0,91** |
|  | thioproline | **1,52** | **1,00** |
