# Supplementary material for: The metabolome of human milk is altered differentially by Holder pasteurization and high hydrostatic pressure processing
Source: Front Nutr. 2023 Feb 20;10:1107054. doi: 10.3389/fnut.2023.1107054 (PMC9987212; doi:10.3389/fnut.2023.1107054)
Supplement: Supplementary file 6 [file Table_6.docx]

**Table S6.** Milk metabolites in lipids metabolism significantly (p≤0.05) modulated in cohort 1 (pooled samples of DM) and cohort 2 (individual samples of DM). DM samples were treated by HoP (HoP) or high hydrostatic pressure (HP) processing. Statistical comparisons were made between the two cohorts. The modulation level is indicated in colored cells (in red: increase; in green: decrease).

| **Sub Pathway** | **Biochemical Name** | **INDIV HP / INDIV HoP** | **HP / HoP** |
| --- | --- | --- | --- |
| Fatty Acid Synthesis | malonate | 0,97 | **0,90** |
| Medium Chain Fatty Acid | caproate (6:0) | **0,77** | **0,80** |
|  | caprylate (8:0) | **0,79** | **0,87** |
|  | caprate (10:0) | **0,91** | 0,90 |
|  | cis-4-decenoate (10:1n6) | **0,37** | **0,81** |
|  | 10-undecenoate (11:1n1) | **0,62** | **0,81** |
|  | 5-dodecenoate (12:1n7) | **0,81** | **0,92** |
| Long Chain Saturated Fatty Acid | myristate (14:0) | 1,13 | **1,33** |
|  | pentadecanoate (15:0) | **0,83** | **1,23** |
| Long Chain Monounsaturated Fatty Acid | myristoleate (14:1n5) | **0,71** | **0,84** |
|  | palmitoleate (16:1n7) | **0,55** | **0,89** |
|  | 10-heptadecenoate (17:1n7) | **0,53** | 0,91 |
|  | oleate/vaccenate (18:1) | **0,53** | 0,89 |
|  | 10-nonadecenoate (19:1n9) | **0,63** | 0,90 |
|  | eicosenoate (20:1) | **0,68** | 0,95 |
| Long Chain Polyunsaturated Fatty Acid | tetradecadienoate (14:2) | **0,53** | 0,92 |
| (n3 and n6) | hexadecatrienoate (16:3n3) | **0,54** | 0,90 |
|  | heptadecatrienoate (17:3) | **0,51** | **0,88** |
|  | stearidonate (18:4n3) | **0,51** | **0,87** |
|  | eicosapentaenoate (EPA; 20:5n3) | **0,52** | 0,92 |
|  | docosapentaenoate (n3 DPA; 22:5n3) | **0,34** | 0,88 |
|  | docosahexaenoate (DHA; 22:6n3) | **0,36** | 1,06 |
|  | hexadecadienoate (16:2n6) | **0,48** | 0,92 |
|  | linoleate (18:2n6) | **0,38** | 0,92 |
|  | linolenate [alpha or gamma; (18:3n3 or 6)] | **0,48** | **0,88** |
|  | dihomo-linoleate (20:2n6) | **0,55** | 0,93 |
|  | dihomo-linolenate (20:3n3 or n6) | **0,42** | 0,89 |
|  | arachidonate (20:4n6) | **0,33** | **0,87** |
|  | adrenate (22:4n6) | **0,48** | 1,00 |
|  | docosapentaenoate (n6 DPA; 22:5n6) | **0,38** | 1,01 |
|  | docosadienoate (22:2n6) | **0,76** | **1,19** |
| Fatty Acid, Branched | (12 or 13)-methylmyristate (a15:0 or i15:0) | **0,80** | 0,73 |
|  | (14 or 15)-methylpalmitate (a17:0 or i17:0) | **0,74** | 1,15 |
| Fatty Acid, Dicarboxylate | azelate (C9-DC) | 0,56 | **0,64** |
|  | sebacate (C10-DC) | **1,75** | 1,21 |
|  | dodecadienoate (12:2) | **0,59** | **0,67** |
| Fatty Acid Metabolism | butyrylcarnitine (C4) | 0,96 | **0,92** |
|  | propionylcarnitine (C3) | 1,00 | **0,89** |
| Fatty Acid Metabolism (Acyl Glycine (AC)) | N-octanoylglycine | **0,75** | 1,09 |
| Fatty Acid Metabolism (AC, Short Chain) | acetylcarnitine (C2) | 0,96 | **0,89** |
| Fatty Acid Metabolism (AC, Medium Chain) | octanoylcarnitine (C8) | **0,87** | **0,74** |
|  | cis-3,4-methyleneheptanoylcarnitine | **0,96** | 0,99 |
|  | laurylcarnitine (C12) | **0,77** | 1,03 |
| Fatty Acid Metabolism (AC, LC Saturated) | palmitoylcarnitine (C16) | 0,99 | **1,21** |
| Fatty Acid Metabolism (AC, Monounsat) | oleoylcarnitine (C18:1) | **0,87** | 1,10 |
| Carnitine Metabolism | deoxycarnitine | **0,91** | 0,96 |
| Fatty Acid, Monohydroxy | 3-hydroxydecanoate | 1,24 | **1,13** |
|  | 3-hydroxylaurate | 1,32 | **1,27** |
|  | 3-hydroxymyristate | 1,37 | **1,35** |
|  | 13-HODE + 9-HODE | **0,68** | **0,89** |
| Phospholipid Metabolism | choline phosphate | **0,89** | **0,87** |
|  | glycerophosphorylcholine (GPC) | **0,93** | **0,94** |
|  | phosphoethanolamine | **0,91** | **0,90** |
|  | glycerophosphoserine | **1,54** | **1,44** |
|  | trimethylamine N-oxide | **0,89** | 1,06 |
| Phosphatidylcholine (PC) | 1-palmitoyl-2-palmitoleoyl-GPC (16:0/16:1) | **0,86** | **0,83** |
|  | 1-palmitoyl-2-oleoyl-GPC (16:0/18:1) | **0,89** | **0,89** |
|  | 1-palmitoyl-2-linoleoyl-GPC (16:0/18:2) | **0,86** | **0,85** |
|  | 1-palmitoyl-2-alpha-linolenoyl-GPC (16:0/18:3n3) | **0,66** | **0,77** |
|  | 1-palmitoyl-2-arachidonoyl-GPC (16:0/20:4n6) | **0,85** | **0,67** |
|  | 1-palmitoyl-2-docosahexaenoyl-GPC (16:0/22:6) | **0,79** | **0,71** |
|  | 1-stearoyl-2-oleoyl-GPC (18:0/18:1) | **0,89** | 0,93 |
|  | 1-stearoyl-2-linoleoyl-GPC (18:0/18:2) | **0,85** | **0,92** |
|  | 1-stearoyl-2-arachidonoyl-GPC (18:0/20:4) | **0,88** | **0,91** |
|  | 1-stearoyl-2-docosahexaenoyl-GPC (18:0/22:6) | **0,85** | 0,93 |
|  | 1-oleoyl-2-linoleoyl-GPC (18:1/18:2) | **0,83** | **0,77** |
|  | 1,2-dilinoleoyl-GPC (18:2/18:2) | **0,80** | **0,60** |
|  | 1-linoleoyl-2-arachidonoyl-GPC (18:2/20:4n6) | 0,90 | **0,47** |
| Phosphatidylethanolamine (PE) | 1-palmitoyl-2-stearoyl-GPE (16:0/18:0) | **1,07** | **1,12** |
|  | 1-palmitoyl-2-oleoyl-GPE (16:0/18:1) | **0,92** | 1,01 |
|  | 1-palmitoyl-2-linoleoyl-GPE (16:0/18:2) | **0,88** | 1,01 |
|  | 1-oleoyl-2-linoleoyl-GPE (18:1/18:2) | **0,87** | **0,92** |
|  | 1,2-dilinoleoyl-GPE (18:2/18:2) | **0,79** | **0,72** |
| Phosphatidylserine (PS) | 1-stearoyl-2-oleoyl-GPS (18:0/18:1) | **1,25** | **1,54** |
|  | 1-stearoyl-2-linoleoyl-GPS (18:0/18:2) | **1,34** | **1,51** |
|  | 1-stearoyl-2-arachidonoyl-GPS (18:0/20:4) | **1,21** | **1,84** |
| Phosphatidylinositol (PI) | 1-stearoyl-2-oleoyl-GPI (18:0/18:1) | 1,05 | **1,13** |
| Lysophospholipid | 1-palmitoyl-GPC (16:0) | **0,68** | **0,76** |
|  | 2-palmitoyl-GPC (16:0) | **1,24** | **1,81** |
|  | 1-stearoyl-GPC (18:0) | **0,87** | **0,76** |
|  | 1-oleoyl-GPC (18:1) | **0,49** | **0,38** |
|  | 1-linoleoyl-GPC (18:2) | **0,28** | **0,31** |
|  | 1-palmitoyl-GPE (16:0) | **0,68** | 1,07 |
|  | 1-oleoyl-GPE (18:1) | **0,40** | **0,34** |
|  | 1-linoleoyl-GPE (18:2) | **0,35** | **0,26** |
|  | 1-stearoyl-GPI (18:0) | **0,73** | **0,89** |
|  | 1-oleoyl-GPI (18:1) | **0,51** | **0,51** |
| Plasmalogen | 1-(1-enyl-stearoyl)-2-oleoyl-GPE (P-18:0/18:1) | **1,08** | **1,12** |
|  | 1-(1-enyl-stearoyl)-2-linoleoyl-GPE (P-18:0/18:2) | 1,02 | **1,14** |
| Monoacylglycerol | 1-myristoylglycerol (14:0) | **0,71** | **0,59** |
|  | 1-pentadecanoylglycerol (15:0) | **0,62** | **0,66** |
|  | 1-palmitoylglycerol (16:0) | **0,62** | **0,67** |
|  | 1-palmitoleoylglycerol (16:1) | **0,33** | **0,49** |
|  | 1-margaroylglycerol (17:0) | **0,54** | **0,75** |
|  | 1-oleoylglycerol (18:1) | **0,40** | **0,56** |
|  | 1-linoleoylglycerol (18:2) | **0,26** | **0,49** |
|  | 1-linolenoylglycerol (18:3) | **0,28** | **0,33** |
|  | 1-dihomo-linolenylglycerol (20:3) | **0,35** | **0,46** |
|  | 1-arachidonylglycerol (20:4) | **0,25** | **0,39** |
|  | 1-docosahexaenoylglycerol (22:6) | **0,30** | **0,67** |
|  | 2-myristoylglycerol (14:0) | **0,90** | **0,77** |
|  | 2-palmitoleoylglycerol (16:1) | **0,25** | **0,32** |
|  | 2-oleoylglycerol (18:1) | 0,45 | **0,52** |
|  | 2-linoleoylglycerol (18:2) | **0,42** | **0,60** |
|  | 1-heptadecenoylglycerol (17:1) | **0,31** | **0,47** |
| Diacylglycerol | diacylglycerol (12:0/18:1, 14:0/16:1, 16:0/14:1) [1] | 1,03 | **0,77** |
|  | diacylglycerol (14:0/18:1, 16:0/16:1) [1] | 0,92 | **0,77** |
|  | diacylglycerol (14:0/18:1, 16:0/16:1) [2] | 1,00 | **0,88** |
|  | diacylglycerol (16:1/18:2 [2], 16:0/18:3 [1]) | **0,61** | 0,89 |
|  | palmitoyl-myristoyl-glycerol (16:0/14:0) [2] | 1,90 | **1,34** |
|  | myristoyl-linoleoyl-glycerol (14:0/18:2) [1] | 0,75 | **0,87** |
|  | palmitoyl-palmitoyl-glycerol (16:0/16:0) [2] | 1,74 | **2,35** |
|  | palmitoleoyl-linoleoyl-glycerol (16:1/18:2) [1] | **0,44** | **0,83** |
|  | palmitoyl-arachidonoyl-glycerol (16:0/20:4) [2] | **0,74** | 0,93 |
|  | palmitoyl-docosahexaenoyl-glycerol (16:0/22:6) [1] | **0,80** | 0,91 |
|  | palmitoyl-docosahexaenoyl-glycerol (16:0/22:6) [2] | **0,93** | **0,88** |
|  | oleoyl-linoleoyl-glycerol (18:1/18:2) [1] | **0,61** | **0,84** |
|  | oleoyl-linoleoyl-glycerol (18:1/18:2) [2] | **0,68** | **0,81** |
|  | linoleoyl-linoleoyl-glycerol (18:2/18:2) [1] | **0,47** | 0,88 |
|  | linoleoyl-linoleoyl-glycerol (18:2/18:2) [2] | **0,56** | 0,96 |
|  | linoleoyl-linolenoyl-glycerol (18:2/18:3) [1] | **0,51** | **0,76** |
|  | linoleoyl-linolenoyl-glycerol (18:2/18:3) [2] | **0,59** | 0,83 |
|  | linolenoyl-linolenoyl-glycerol (18:3/18:3) [1] | **0,55** | **0,68** |
|  | linolenoyl-linolenoyl-glycerol (18:3/18:3) [2] | **0,68** | **0,60** |
|  | oleoyl-arachidonoyl-glycerol (18:1/20:4) [1] | **0,59** | **0,76** |
|  | oleoyl-arachidonoyl-glycerol (18:1/20:4) [2] | **0,71** | **0,79** |
|  | linoleoyl-arachidonoyl-glycerol (18:2/20:4) [1] | **0,28** | **0,68** |
|  | linoleoyl-arachidonoyl-glycerol (18:2/20:4) [2] | **0,36** | **0,76** |
|  | stearoyl-docosahexaenoyl-glycerol (18:0/22:6) [2] | **0,83** | **0,91** |
|  | linoleoyl-docosahexaenoyl-glycerol (18:2/22:6) [1] | **0,28** | **0,73** |
|  | linoleoyl-docosahexaenoyl-glycerol (18:2/22:6) [2] | **0,34** | **0,81** |
| Galactosyl Glycerolipids | galactosylglycerol | **0,93** | **1,17** |
| Dihydroceramides | N-palmitoyl-sphinganine (d18:0/16:0) | **1,27** | **1,22** |
| Ceramides | N-palmitoyl-sphingosine (d18:1/16:0) | **1,46** | 1,15 |
|  | N-palmitoyl-sphingadienine (d18:2/16:0) | **1,70** | **1,85** |
|  | N-behenoyl-sphingadienine (d18:2/22:0) | **1,57** | 1,12 |
|  | ceramide (d18:1/14:0, d16:1/16:0) | **2,16** | **1,40** |
|  | ceramide (d16:1/24:1, d18:1/22:1) | **1,94** | **1,57** |
|  | ceramide (d18:2/24:1, d18:1/24:2) | **1,66** | **1,49** |
| Dihydrosphingomyelins | myristoyl dihydrosphingomyelin (d18:0/14:0) | **0,80** | 0,95 |
|  | palmitoyl dihydrosphingomyelin (d18:0/16:0) | **0,90** | 0,96 |
|  | behenoyl dihydrosphingomyelin (d18:0/22:0) | **0,83** | 1,01 |
| Sphingomyelins | palmitoyl sphingomyelin (d18:1/16:0) | **0,91** | 0,91 |
|  | hydroxypalmitoyl sphingomyelin (d18:1/16:0(OH)) | **0,92** | **0,85** |
|  | stearoyl sphingomyelin (d18:1/18:0) | **0,89** | 0,95 |
|  | behenoyl sphingomyelin (d18:1/22:0) | **0,88** | 1,00 |
|  | tricosanoyl sphingomyelin (d18:1/23:0) | **0,88** | 1,00 |
|  | lignoceroyl sphingomyelin (d18:1/24:0) | **0,87** | 0,98 |
|  | sphingomyelin (d18:1/14:0, d16:1/16:0) | **0,83** | 0,93 |
|  | sphingomyelin (d18:2/14:0, d18:1/14:1) | **0,86** | 0,95 |
|  | sphingomyelin (d18:2/16:0, d18:1/16:1) | **0,89** | **0,79** |
|  | sphingomyelin (d18:1/17:0, d17:1/18:0, d19:1/16:0) | **0,90** | 0,89 |
|  | sphingomyelin (d18:1/20:0, d16:1/22:0) | **0,91** | 0,97 |
|  | sphingomyelin (d18:1/20:1, d18:2/20:0) | **0,87** | 0,90 |
|  | sphingomyelin (d18:1/21:0, d17:1/22:0, d16:1/23:0) | 0,97 | **0,88** |
|  | sphingomyelin (d18:1/22:1, d18:2/22:0, d16:1/24:1) | **0,90** | 0,94 |
| Sterol | 4-cholesten-3-one | **0,82** | 0,90 |
