# Supplementary material for: The metabolome of human milk is altered differentially by Holder pasteurization and high hydrostatic pressure processing
Source: Front Nutr. 2023 Feb 20;10:1107054. doi: 10.3389/fnut.2023.1107054 (PMC9987212; doi:10.3389/fnut.2023.1107054)
Supplement: Supplementary file 7 [file Table_7.docx]

**Table S7.** Milk metabolites in nucleotides metabolism significantly (p≤0.05) modulated in cohort 1 (pooled samples of DM) and cohort 2 (individual samples of DM). DM samples were treated by HoP (HoP) or high hydrostatic pressure (HP) processing. Statistical comparisons were made between the two cohorts. The modulation level is indicated in colored cells (in red: increase; in green: decrease).

| **Sub Pathway** | **Biochemical Name** | **INDIV HP / INDIV HoP** | **HP / HoP** |
| --- | --- | --- | --- |
| Purine Metabolism, | AICA ribonucleotide | **0,80** | **0,86** |
| (Hypo)Xanthine/Inosine containing | inosine | 0,94 | **1,73** |
|  | allantoin | 0,94 | **0,81** |
| Purine Metabolism, Adenine containing | adenosine 5'-monophosphate (AMP) | **1,47** | **2,08** |
|  | adenosine 2'-monophosphate (2'-AMP) | **1,97** | **4,31** |
|  | adenosine | 0,48 | **3,67** |
|  | adenine | **0,26** | **0,23** |
|  | N6-methyladenosine | **0,49** | **0,44** |
|  | N6-carbamoylthreonyladenosine | 1,00 | **0,91** |
| Purine Metabolism, Guanine containing | guanosine | 2,01 | **2,75** |
|  | guanine | **1,74** | **1,67** |
|  | N2,N2-dimethylguanosine | 1,04 | **1,22** |
| Pyrimidine Metabolism, Uracil containing | uridine 5'-monophosphate (UMP) | **0,88** | **1,16** |
|  | uridine | 1,08 | **1,08** |
|  | 3-(3-amino-3-carboxypropyl)uridine | 1,00 | **0,89** |
| Pyrimidine Metabolism, Cytidine containing | cytidine 5'-monophosphate (5'-CMP) | **0,52** | **0,60** |
|  | cytidine 2' or 3'-monophosphate | **0,60** | **0,71** |
|  | cytidine | 1,20 | **1,51** |
|  | 2'-deoxycytidine | 1,11 | **1,61** |
| Dinucleotide | (3'-5')-adenylylcytidine | 1,39 | **1,83** |
|  | (3'-5')-adenylyluridine | 1,85 | **1,85** |
|  | (3'-5')-cytidylyluridine | **1,87** | **1,91** |
