# Supplementary material for: The metabolome of human milk is altered differentially by Holder pasteurization and high hydrostatic pressure processing
Source: Front Nutr. 2023 Feb 20;10:1107054. doi: 10.3389/fnut.2023.1107054 (PMC9987212; doi:10.3389/fnut.2023.1107054)
Supplement: Supplementary file 8 [file Table_8.docx]

**Table S8.** Milk metabolites in amino acids metabolism significantly (p≤0.05) modulated in cohort 1 (pooled samples of DM) and cohort 2 (individual samples of DM). DM samples were treated by HoP (HoP) or high hydrostatic pressure (HP) processing. Statistical comparisons were made between the two cohorts. The modulation level is indicated in colored cells (in red: increase; in green: decrease).

| **Sub Pathway** | **Biochemical Name** | **INDIV HP / INDIV HoP** | **HP / HoP** |
| --- | --- | --- | --- |
| Glycine, Serine and Threonine Metabolism | N-acetylglycine | 0,90 | **1,27** |
|  | 3-phosphoserine | **0,79** | 0,71 |
|  | threonine | 0,96 | **0,91** |
| Alanine and Aspartate Metabolism | aspartate | **1,07** | 1,01 |
| Glutamate Metabolism | glutamine | **0,36** | 0,79 |
| Histidine Metabolism | 3-methylhistidine | **0,84** | 1,04 |
|  | trans-urocanate | **0,86** | 1,06 |
|  | 1-ribosyl-imidazoleacetate | **0,70** | 0,86 |
| Lysine Metabolism | lysine | **1,61** | **1,59** |
| Tyrosine Metabolism | phenol sulfate | 0,91 | **0,88** |
| Leucine, Isoleucine and Valine Metabolism | leucine | **0,86** | 0,99 |
|  | 3-methyl-2-oxobutyrate | 0,93 | **0,86** |
|  | alpha-hydroxyisovalerate | 0,98 | **0,86** |
| Methionine, Cysteine, SAM | N-formylmethionine | **0,78** | 0,94 |
| and Taurine Metabolism | methionine sulfone | **0,88** | 1,17 |
|  | S-adenosylmethionine (SAM) | **1,41** | **1,61** |
|  | cysteine | **0,48** | **0,56** |
|  | S-methylcysteine | **0,83** | **0,92** |
|  | cysteine s-sulfate | **0,65** | **0,56** |
|  | hypotaurine | **1,26** | **0,84** |
| Urea cycle; Arginine and Proline Metabolism | arginine | **1,34** | **1,27** |
|  | 3-amino-2-piperidone | **0,86** | 0,98 |
|  | homocitrulline | **0,90** | 1,13 |
| Creatine Metabolism | creatine | 0,95 | **0,86** |
|  | creatine phosphate | **1,46** | **1,53** |
| Polyamine Metabolism | 5-methylthioadenosine (MTA) | **0,31** | **0,44** |
| Glutathione Metabolism | cysteinylglycine disulfide | **0,59** | **0,61** |
|  | cys-gly, oxidized | **0,90** | **0,46** |
|  | 5-oxoproline | **0,82** | 0,86 |
|  | 2-hydroxybutyrate/2-hydroxyisobutyrate | 0,96 | **0,82** |
| Dipeptide | cyclo(leu-pro) | **0,62** | 1,18 |
| Modified Peptides | N,N-dimethyl-pro-pro | 1,02 | **0,88** |
